# Expression of myo-inositol cotransporters in the sciatic nerve and dorsal root ganglia in experimental diabetes

**DOI:** 10.1590/1414-431X20198589

**Published:** 2019-06-03

**Authors:** V.X. Farias, P.N. Uchoa, C.P. Aquino, L.R.G. Britto, M.C. Fonteles, J.H. Leal-Cardoso, K.S. Silva-Alves, A. Havt, M.M.G. Prata, D.B. Heimark, N.R.F. Nascimento, C.F. Santos

**Affiliations:** 1 Instituto Superior de Ciências Biomédicas, Universidade Estadual do Ceará, Fortaleza, CE, Brasil; 2 Instituto de Ciências Biomédicas, Laboratório de Neurofisiologia, Universidade de São Paulo, SP, Brasil; 3 Instituto de Ciências Biomédicas, Universidade Federal do Ceará, Fortaleza, CE, Brasil

**Keywords:** Diabetic neuropathy, Inositol metabolism, Peripheral nervous tissues

## Abstract

The transport of myo-inositol is the main mechanism for the maintenance of its high intracellular levels. We aimed to measure the mRNA and protein levels of myo-inositol cotransporters in the sciatic nerve (SN) and dorsal root ganglia (DRG) during experimental diabetes. Streptozotocin-induced (STZ; 4, 8, and 12 weeks; 65 mg/kg; *ip*) diabetic rats (DB) and age-matched euglycemic (E) rats were used for the analysis of mRNA and protein levels of sodium myo-inositol cotransporters 1, 2 (SMIT1, SMIT2) or H^+^/myo-inositol cotransporter (HMIT). There was a significant reduction in the mRNA levels for SMIT1 in the SN and DRG (by 36.9 and 31.0%) in the 4-week DB (DB4) group compared to the E group. SMIT2 was not expressed in SN. The mRNA level for SMIT2 was up-regulated only in the DRG in the DB4 group. On the other hand, the protein level of SMIT1 decreased by 42.5, 41.3, and 44.8% in the SN after 4, 8, and 12 weeks of diabetes, respectively. In addition, there was a decrease of 64.3 and 58.0% of HMIT in membrane and cytosolic fractions, respectively, in the SN of the DB4 group. In the DRG, there was an increase of 230 and 86.3% for SMIT1 and HMIT, respectively, in the DB12 group. The levels of the main inositol transporters, SMIT1 and HMIT, were greatly reduced in the SN but not in the DRG. SMIT-1 was selectively reduced in the sciatic nerve during experimental STZ-induced diabetes.

## Introduction

Myo-inositol is a natural cyclitol that is mostly obtained from diet but is also synthesized in the kidney. This compound is an organic osmolyte protecting cells from osmotic stress and a precursor of inositolphosphoglycans, phosphatidylinositols, and phosphoinositides that are involved in signal transduction (1). For instance, myo-inositol is found as free myo-inositol or bound covalently to phospholipids, as the structural basis for a number of secondary messengers, including inositol triphosphates, phosphatidylinositol (PI), and polyphosphoinositides (i.e., PI(4)P, PI(4,5)P2, and PI(3,4,5)P3). The synthesis of PI requires a relatively high K_m_ (1.5–2.5 mM) making intracellular myo-inositol homeostasis very important to numerous cell activities (2,3).

Myo-inositol and D-chiro-inositol are also components of glycosyl-phosphatidylinositol anchors and inositol phosphoglycans, which are second messengers of insulin action (2,3). Myo-inositol has been shown to improve glucose tolerance in both animals and humans (1,4,5).

Three distinct mechanisms maintain intracellular concentrations of myo-inositol: i) inositol transport across the plasma membrane via a specific carrier molecule; ii) *de novo* synthesis of myo-inositol from D-glucose-6-phosphate; iii) efflux of myo-inositol, which is mediated by a volume-sensitive organic osmolyte anion channel (6).

Myo-inositol can be transported from the extracellular medium by three carriers identified so far: the sodium-myo-inositol transporters 1 and 2 (SMIT1 and SMIT2) and H^+^/myo-inositol transporter (HMIT). SMIT1 is a protein with 43% sequence similarity to SMIT2 and both are expressed in the brain and in some peripheral tissues such as liver, kidney, intestine, and skeletal muscle (7,8). HMIT is predominantly expressed in the central nervous system, especially in the brain. The rate of activity of this cotransporter is optimum at low pH (∼5). Furthermore, the expression of HMIT on the membrane surface is dependent on cell depolarization, PKC activation, and increased intracellular calcium, while the expression of both SMIT1 and SMIT2 are constitutive (9).

Disturbances in the metabolism of inositol have been postulated as an underlying mechanism for the development of diabetic neuropathy (10,11). The level of myo-inositol is decreased as early as one week after the induction of experimental diabetes, and after four weeks of diabetes it is reduced by about 65% (12,13). Transport of myo-inositol from the extracellular medium into the cells is the main mechanism involved in the maintenance of the high intracellular levels of myo-inositol. The myo-inositol level in mammalian tissues ranges from 0.1 to 16 mM (14,15). It is high in adult brain (millimolar level) and is reduced by 96% in Solute Carrier Family 5 Member 3 (slc5A3) gene knockout mice. This gene codes for SMIT1, and slc5A3 knockout mice need to be maintained on myo-inositol supplementation throughout their life (8,16).

Therefore, changes in the expression of such transporters may have a role in the pathogenesis of diabetic neuropathy. The aim of this study was to investigate and compare both the mRNA and protein expression of myo-inositol transporters in the peripheral nervous system during experimental diabetes.

## Material and Methods

### Animals and induction of diabetes

Male rats (*Rattus norvegicus*), with free access to food and water, and weighing 180–250 g, were injected intraperitoneally with streptozotocin (STZ; 65 mg/kg) diluted in 0.1M citrate buffer (pH 4.0). Euglycemic control animals received an equal volume of citrate buffer. All the protocols were approved by the Committee for Care and Use of Animals in Research of the Ceará State University under protocol #11518153-9/68. Diabetes was verified using blood glucose monitoring strips. Animals with blood glucose levels above 200 mg/dL were included in this study. In order to check for sciatic nerve (SN) function, we evaluated chronaxy, rheobase, conduction velocity, and nerve growth factor (NGF) expression levels. After each experimental period, time-matched euglycemic (E) or 4, 8, or 12 week-long diabetic rats (DB) (E4, E8, E12, DB4, DB8, and DB12, respectively) were sacrificed, and SN and dorsal root ganglia (DRG) harvested for analysis of slc5A3 (SMIT1), slcA11 (SMIT2), and slc2A13 (HMIT) mRNA expression by RT-PCR and protein levels by western blot analyses.

### Sciatic nerve function analysis

#### Extracellular recording

Briefly, the SN was mounted in a moist chamber, and one of its ends was stimulated with a S48 electrical stimulator (Grass Instruments Co., USA). The electrical stimulation of the SN promotes a depolarization wave, which propagates along axons in the SN, known as evoked compound action potential (CAP). The CAP was recorded with platinum electrodes placed 40–50 mm from the stimulating electrodes and continuously monitored using an oscilloscope (Model 547, Tektronix, Inc., USA). Computer acquisition hardware was used for data storage and analysis as well as AxoScope software (Axon Instruments, Inc., USA). A 15–20-mm segment of SN was suspended between stimulation and recording electrodes and immersed in a modified Locke’s solution. In order to register the electrical properties and parameters related to excitability, SN was allowed to stabilize for a period of 30 min to 2 h, until stable peak-to-peak CAP amplitude (i.e., a stable difference between maximum positive and negative amplitudes of CAP) was achieved. The parameter measured in evoked CAP was the conduction velocity of CAP components. Strength-duration curves with voltage square wave stimulation were used to determine rheobase and chronaxy. Rheobase is defined as the threshold stimulus voltage for an active response with a long duration pulse (1000 µs) and chronaxy is the threshold duration for an active response with a stimulus twice the rheobase.

### RT-PCR

Gene expression of SMIT1, SMIT2, and HMIT were evaluated using a CFX96 touch detection system (Bio-Rad, USA). Tyrosine 3-monooxygenase/tryptophan 5-monoxygenase activation protein zeta polypeptide (*YWHAζ*) was used as the reference gene. Real-time PCR assays were performed in a final volume of 25 µL iQSYBR green supermix (Bio-Rad), 200 nM (each) primers, and 1 µL cDNA from the sample. The PCR conditions were as follows: an initial denaturation period of 7 min at 95°C followed by 39 cycles of gene amplification. Each cycle consisted of an initial denaturation step of 20 s at 95°C, followed by an annealing step of 20 s at 60°C and an extension step of 45 s at 72°C. The samples were then subjected to an extension step of 3 min at 72°C. To measure the specificity of the applied amplifications, we performed a melting curve analysis. Gene expression was obtained by applying the mathematical 2^-ΔΔCt^ method.

### Western immunoblot analyses

SN and DRG samples were homogenized in buffer A (10 mM Tris-HCl, 250 mM saccharose, 2 mM EDTA, 0.1% 2 mM EGTA, 1000 U/mL aprotinin, 0.8 µg/mL leupeptin, 2 mM PMSF) using TissueLyser (Qiagen, Germany). The resulting homogenates were centrifuged at 17,000 *g* at 4°C for 30 min and the supernatant of this procedure was taken as the cytosolic fraction. The resulting pellet was resuspended in lysis buffer (20 mM HEPES/NaOH, 150 mM NaCl, 1% Triton X-100, 10% glycerol, 8 mM EGTA, 15 mM MgCl_2_, 2 mM PMSF), centrifuged at 17,000 *g* at 4°C for 30 min, and the supernatant of this procedure was taken as the membrane fraction (17). The levels of HMIT, myo-inositol phosphate synthase (MIPS), myo-inositol oxygenase (MIOX), and neural growth factor (NGF) protein expression were measured in the cytosolic fraction and the membrane fraction was used for the evaluation of the expression of SMIT1, SMIT2, and HMIT.

Proteins were determined using SDS-PAGE gel electrophoresis followed by imunnoblotting. Briefly, aliquots of 50 µg (DRG) or 75 µg (SN) protein was subjected to SDS-PAGE (10% acrylamide) and transferred to a PVDF membrane at 500 mA overnight using a Hoefer apparatus (Holliston, USA). Thereafter, the membrane was immersed in a TBS-T blocking solution (0.05% Tween 20/0.1 M Tris/0.15 M NaCl, pH 7.5, plus 5% BSA). The membranes were then incubated overnight with the primary antibody for NGF, MIPS, MIOX (1:100, Santa Cruz Biotechnology^®^, USA), SMIT1, SMIT2, HMIT (1:1000, GenOne Biotechnologies, Brazil), or β-actin (internal loading control) (1:5000, Sigma, USA). The membrane was further incubated for 1 h at room temperature with an anti-rabbit secondary antibody conjugated with alkaline phosphatase. The blot was developed using a chemiluminescence kit (CDP star, Applied Biosystem, USA) and read using a Chemidoc XRS+ photodocumentation system (Bio-Rad).

### Statistical analysis

Statistical analysis was performed using unpaired Student’s *t*-test for comparison between groups against age-matched euglycemic controls.

## Results

### Plasma glucose concentrations and weights

Diabetes caused an approximate four-fold increase in plasma glucose concentrations (P<0.05) in the DB4, DB8, and DB12 groups. The body weight of DB12 rats was significantly (P<0.05) lower than that of E rats (Table 1).

**Table 1:** Changes in body weight and blood glucose levels of diabetic and control rats.

Groups	Body weight (g)		Blood glucose (mg/dL)
	Control	End of experimental period	End of experimental period
E (n=14)	209.20±6.41	299.00±12.20^a^	105.9±8.37
DB4 (n=8)	196.90±4.62	209.10±9.13^b^	507.8±22.20^b^
DB8 (n=11)	196.40±3.84	220.0±12.91^b^	466.9±30.34^b^
DB12 (n=9)	202.80±8.82	228.0±12.57^b^	540.6±17.05^b^

Data are reported as means±SE. DB4, DB8, DB12: 4-, 8-, and 12-week diabetic rats; E: euglycemic rats. n: number of animals studied in each group. ^a^P<0.001 *vs* control; ^b^P<0.001 *vs* E group (Student’s *t*-test).

### Excitability and electrical properties

As shown in Table 2, there was a significant decrease in excitability of the SN of diabetic animals. Two parameters related to excitability were measured: rheobase and chronaxy. The rheobase of the DB8 and DB12 groups had a significant increase of 7.9% and 14.4%, respectively, compared to rheobase that was observed in the respective E groups (3.4±0.09 V and 3.45±0.09 V *vs* 3.01±0.09 V and 3.15±0.06 V, respectively). Chronaxy values were increased in the DB4, DB8, and DB12 groups (63.88±2.6, 55.64±1.6, and 61.71±4.5 μs, respectively) compared to the respective E groups E4, E8, and E12 (48.33±2.6, 50.08±1.5, and 50.85±4.5 μs, respectively).

**Table 2:** Excitability and electrical properties of euglycemic groups (E4, E8, and E12) and diabetic (DB4, DB8, and DB12) groups at 4, 8, and 12 weeks.

Groups	1st component conduction velocity (m/s)	2nd component conduction velocity (m/s)	Rheobase (V)	Chronaxy (µs)
E4	106.80±6.06 (n=14)	44.38±3.57 (n=14)	3.26±0.07 (n=15)	48.33±2.68 (n=12)
DB4	87.65±5.15 (n=16)^a^	36.04±2.90 (n=16)	3.46±0.11 (n=16)	63.88±2.27 (n=16)^a^
E8	119.9±5.06 (n=11)	42.66±2.63 (n=12)	3.15±0.06 (n=12)	50.08±1.51 (n=12)
DB8	96.65±5.03 (n=14)^b^	35.49±2.65 (n=14)	3.40±0.09 (n=14)^b^	55.64±2.27 (n=14)^b^
E12	129.9±4.93 (n=12)	53.10±3.23 (n=13)	3.01±0.06 (n=13)	50.85±2.12 (n=13)
DB12	104.5±3.12 (n=22)^c^	39.81±1.73 (n=24)^c^	3.45±0.09 (n=24)^c^	61.71±4.58 (n=24)

Data are reported as means±SE. n: number of nerves studied in each group. ^a^P<0.05 *vs* E4; ^b^P<0.05 *vs* E8; ^c^P<0.05 *vs* E12 (Student’s *t*-test).

The current analysis showed that the conduction velocity of 1st CAP of the SN in the animals of DB4, DB8, and DB12 groups (87.75±5.1, 96.65±5.0, and 104.50±3.1 m/s, respectively) was significantly decreased by 17.9, 19.4, and 19.5% (P<0.05) compared to their respective E4, E8, and E12 controls (106.8±6.0, 119.9±5.0, and 129.9±4.9 m/s, respectively). The conduction velocity of the 2nd compound action potential of the SN in the DB12 group was 39.81±1.7 m/s, while in the E control, it was 53.1±3.2 m/s (P<0.05).

### NGF expression in the sciatic nerve of diabetic rats

**Figure 1:**
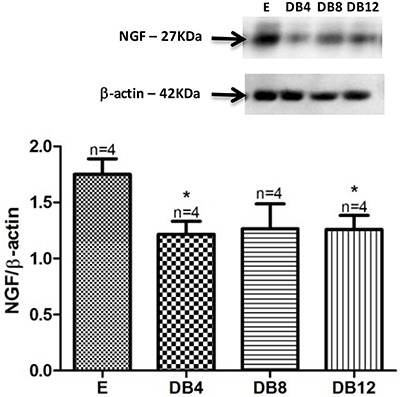
Nerve growth factor (NGF) protein expression in rats with 4, 8, and 12 weeks in diabetic state (DB4, DB8, and DB12, respectively) compared to euglycemic rats (E). β-actin was used as loading control. Data are reported as means±SE. *P<0.05 *vs* E group (Student’s *t*-test).

The protein expression level of NGF showed a significant decrease of 30.8% in the DB4 group and 28.5% in the DB12 group, respectively, compared to the euglycemic group (Figure 1).

### MIPS and MIOX expression in the DRG and SN

The protein expression of both MIPS and MIOX, which are involved with myo-inositol synthesis and oxidation, respectively, was not changed in the diabetic model studied.

### Myo-inositol transporters in the peripheral nervous system

**Figure 2:**
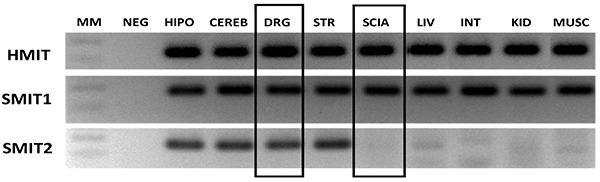
Sodium myo-inositol cotransporters 1 and 2 (SMIT1, SMIT2), and H+/myo-inositol cotransporter (HMIT) detection by regular PCR and ethidium bromide-stained agarose gels on mRNA extracted from sciatic nerve (SCIA) and dorsal root ganglia (DRG), hippocampus (HIPO), cerebellum (CEREB), striatum (STR), liver (LIV), intestine (INT), kidney (KID), and skeletal muscle (MUSC). Total RNA (1000 ng) was loaded into each lane, except for SCIA (250 ng) and DRG (500 ng).

The first study we performed was a qualitative PCR analysis in samples of SN, DRG, hippocampus, cerebellum, striatum, liver, intestine, kidney, and skeletal muscle to check whether those tissues expressed the myo-inositol transporters. SMIT1 was expressed in all tissues studied. Remarkably, we have found for the first time that SMIT2 was not expressed in rat SN, and that HMIT was abundantly expressed in both the SN and the DRG (Figure 2).

### Myo-inositol cotransporters relative gene and protein expression

**Figure 3:**
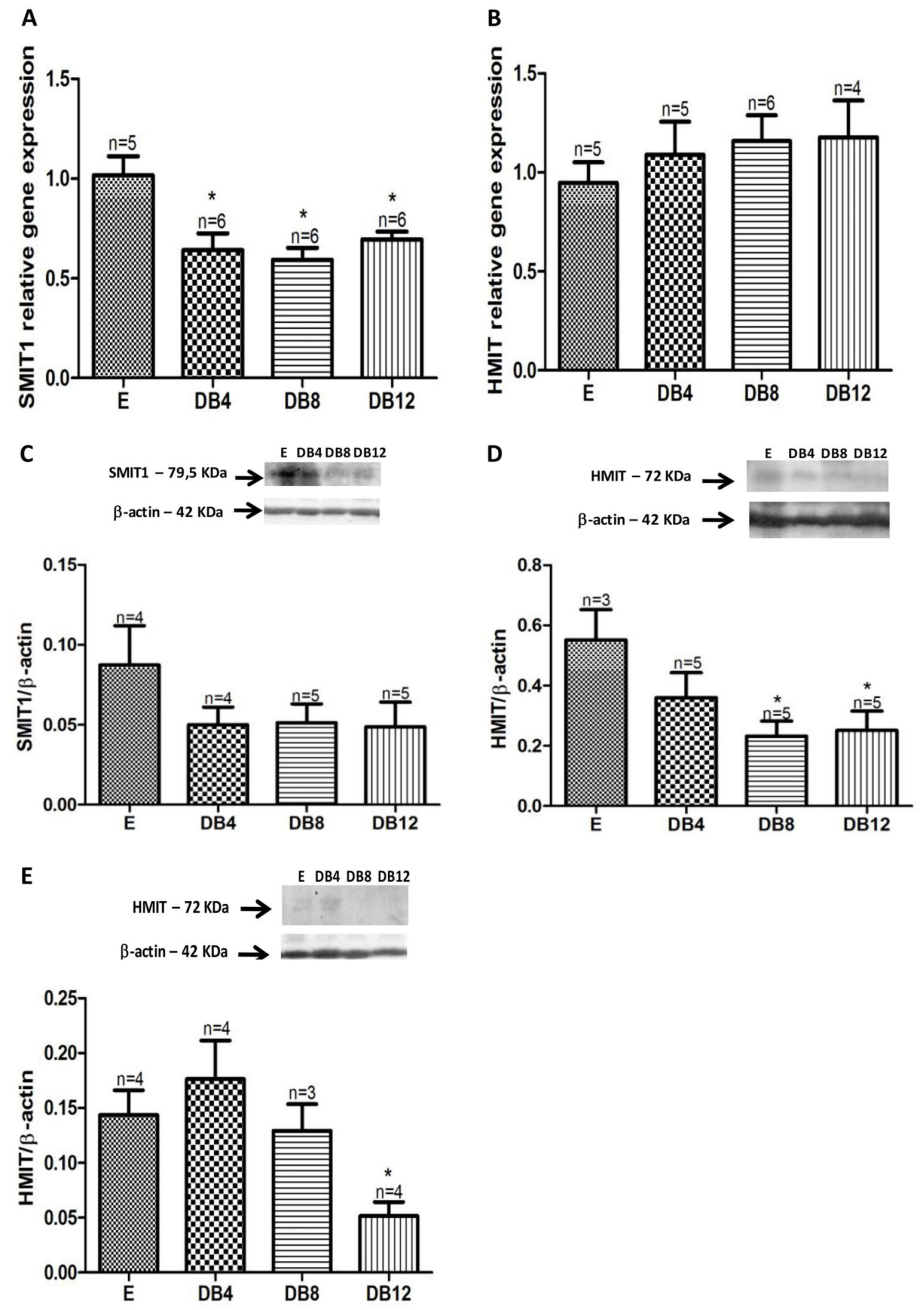
Relative quantitative mRNA expression of the sodium myo-inositol cotransporter (SMIT1) and H+/myo-inositol cotransporter (HMIT) in the sciatic nerve (**A**, **B**) of 4, 8, and 12 week diabetic rats (DB4, DB8, DB12, respectively). **C**, SMIT1 protein expression in the sciatic nerve membrane fraction in diabetic rats compared to euglycemic rats (E) rats. HMIT protein expression in the sciatic nerve cytoplasmic (**D**) and membrane fraction (**E**) in diabetic rats compared to E. β-actin detection was used as loading control. Data are reported as means±SE. *P<0.05 *vs* E (Student’s *t*-test).

In the SN, a significant reduction of SMIT1 mRNA expression in the DB4, DB8, and DB12 groups (36.9, 41.5, and 33.6%, respectively) was found. No significant alterations of HMIT gene expression was observed in this model of STZ-induced diabetes. On the other hand, the protein expression of SMIT1 was reduced by 42.52, 41.37, and 44.82% in the SN of DB4, DB8, and DB12 groups, respectively. The expression of HMIT in the cytoplasmic SN fraction was significantly lower in DB8 (–58%) and DB12 (–45.5%) groups compared to the E animals. In membrane fractions of the SN, HMIT expression was decreased by 64.3% in the DB12 group compared to E controls (P<0.05) (Figure 3).

**Figure 4:**
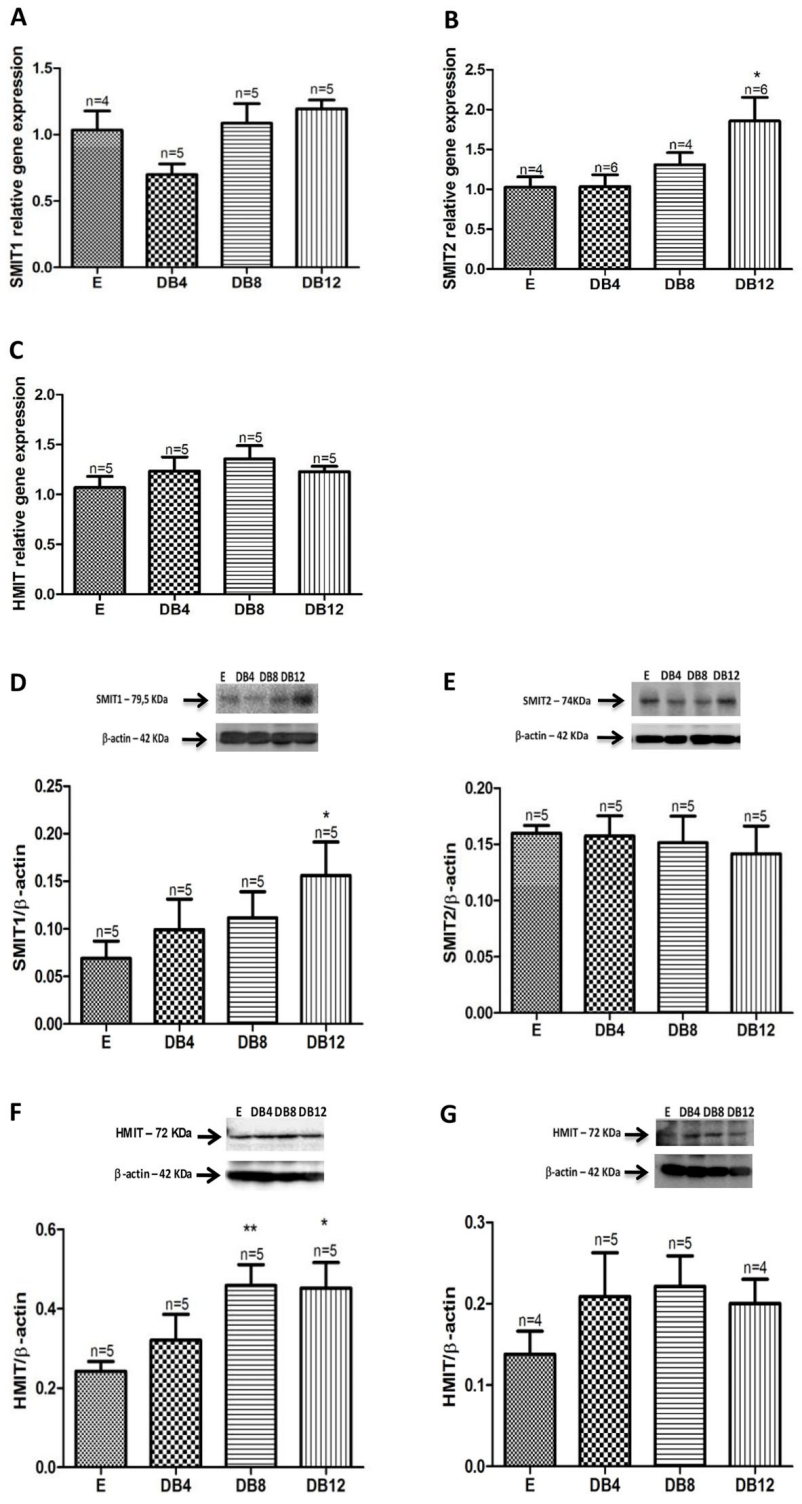
Relative quantitative mRNA expression of the sodium myo-inositol cotransporters 1, 2, and H+/myo-inositol cotransporter (SMIT1, SMIT2, and HMIT, **A**, **B**, **C**, respectively) in the dorsal root ganglia (DRG) of 4, 8, and 12 week diabetic rats (DB4, DB8, DB12, respectively) and euglycemic rats (E). SMIT1 (**D**) and SMIT2 (**E**) protein expression in DRG membrane fraction of diabetic rats compared to DRG E rats. HMIT protein expression in the DRG cytoplasmic (**F**) and membrane fraction (**G**) of diabetic rats compared to the sciatic nerve of E rats. β-actin detection was used as a loading control. Data are reported as means±SE. *P<0.05 *vs* E rats (Student’s *t*-test).

No significant difference in the mRNA level of SMIT1 in the DRG was found between the euglycemic and diabetic control groups. In contrast, SMIT2 showed 81.3% greater expression in the DB12 group compared to the euglycemic group. In this tissue, there was no significant change in HMIT expression in STZ-induced diabetes. In the DRG, SMIT1 showed 2.26-fold greater expression in the DB12 group compared to the E control group (0.156±0.03 *vs* 0.069±0.01, P<0.05) (Figure 4). On the other hand, the expression of SMIT2 did not change in STZ-induced diabetes. There was a significant elevation in HMIT expression in the cytoplasmic fraction of DRG in the DB8 and DB12 groups compared to the E controls (increase of 89.25 and 86.36 %, respectively). In the membrane fraction of the dorsal root ganglia, we observed a trend toward enhancement in HMIT expression in all DB groups studied.

## Discussion

The intracellular concentration of myo-inositol has been consistently found to be decreased during STZ-induced diabetes in both experimental animals (13,18,19) and human subjects (20,21). Decreased levels of myo-inositol in nerves are always correlated with neuronal dysfunction (22). Myo-inositol is a precursor of important metabolites such as phosphoinositols. It is also an important organic osmolyte that helps maintain intracellular homeostasis during osmotic stress due to chronic hyperglycemia (23).

The administration of D-chiro-inositol, an epimer of myo-inositol, improves both somatic and autonomic nerve functions and preserves nerve conduction velocity and myelination in sciatic nerves of STZ-diabetic mice (24).

Therefore, we hypothesized that the transport of myo-inositol via both SMIT1 or 2 and HMIT is compromised during STZ-induced diabetes.

In the STZ-induced diabetes model used herein, both chronaxy and rheobase were increased in the SN. The tissue was less excitable compared to the nerves of E controls. Similar findings were reported for chronaxy and rheobase of the SN of the rats with diabetes induced in the neonatal period (25). Furthermore, the nerve conduction velocity was decreased in our model as early as four weeks after diabetes induction. This data is consistent with the findings of others (12,13,26). In addition, we found decreased levels of NGF in SN of the diabetic animals, which is also consistent with the findings of others (27,28).

There are few studies on the mRNA expression of myo-inositol transporters in different tissues. The qualitative PCR analysis performed herein confirmed the expression of SMIT1 mRNA in the cerebral cortex, hippocampus, and cerebellum (29), intestine (30), and kidney (31,32). In the present work, we found that the mRNA for SMIT1 and HMIT but not SMIT2 was expressed in the SN. In addition, in the DRG all three transporters were expressed, SMIT2 being the least abundant. As far as we know, this is the first demonstration of HMIT expression in the SN and DRG, and of SMIT1 expression in the DRG. In addition, this is the first demonstration of the presence of SMIT2 in the DRG and striatum.

The mRNA levels for SMIT1 were decreased in the SN, four weeks after the onset of diabetes and this alteration was maintained in DB8 and DB12 groups. On the other hand, the mRNA expression for HMIT did not change in any of the groups studied.

On the other hand, the protein expression level for both SMIT1 and HMIT was remarkably decreased in the SN obtained from DB4 animals. The expression of HMIT in the membrane fraction was significantly decreased only after 12 weeks, but in the cytoplasmic fractions, the content of HMIT was diminished in both the DB8 and DB12 group. HMIT belongs to the class III GLUT-family of proteins and is primarily stored in cytoplasmic vesicles and translocated to the membrane in response to stimuli, such as increased intracellular calcium levels, decreased intracellular pH, or increased PKC activity (9). Therefore, we suggest that in the chronic setting of diabetes, the membrane pool could also be affected because the cytoplasmic pool was decreased earlier in STZ-induced diabetes.

In the DRG, the mRNA levels for SMIT1 and HMIT were not modified in any of the groups or periods studied. The mRNA levels for SMIT2 were increased only in the tissues obtained from DB12 rats. The protein expression of SMIT1 was increased in the DB12 group while the expression of SMIT2 did not change during STZ-induced diabetes. The levels of HMIT protein was increased in the cytoplasmic pool but not in the membrane fraction. Therefore, the main finding was that in the DRG, the expression of the main transporters, i.e. SMIT1 and HMIT, were not diminished, the opposite of what we found in the SN.

The expression of SMIT2 in the DRG, but not in the SN, and the increased expression of SMIT1 in DRG could probably counter-regulate the increased metabolic and osmotic stress in the DRG soma during STZ-induced diabetes. For instance, Llewelyn and collaborators showed a slight decrease in the content of myo-inositol in the DRG in one- and two-week STZ-diabetic rats that restored to normal eight weeks after the onset of diabetes (35).

On the other hand, the lack of SMIT2 expression in the SN and decreased expression of both SMIT1 and HMIT are probably related to the decreased concentration of myo-inositol shown by others (12,13,18,33,34).

A major limitation of the present study is that we were not able to measure myo-inositol concentrations in the SN or DRG due to technical limitations. Therefore, a correlation between decreased levels of myo-inositol transporters and myo-inositol concentrations was not possible. However, by using the same experimental approach, a decreased content of myo-inositol in peripheral nerves has been consistently reported (33,34). Remarkably, the content of myo-inositol in the rat SN has been shown to be reduced by more than 50% after 4 weeks of STZ-diabetes (12,13). In addition, Gillon and Hawthorne (1983) have shown that the SN myo-inositol content is decreased by as long as 16 weeks after diabetes induction (18).

The administration of myo-inositol, D-chiro-inositol, pinitol, and especially dibutyryl-D-chiro-inositol can restore the function of both autonomic and somatic nerves in STZ-induced diabetic neuropathy (20,21,24,36,37).

For instance, our group has shown that the treatment of diabetic mice with D-chiro-inositol, an epimer of myo-inositol, can restore nerve conduction velocity and preserve nerve structural integrity (24) and that D-chiro-inositol, methyl-D-chiro-inositol, and dibutyryl-D-chiro-inositol can prevent and ameliorate somatic and autonomic nerve function in rats and rabbits (37).

In addition, the chronic inhibition of myo-inositol transport with L-fucose induces a neuropathy that resembles diabetic neuropathy resulting in decreased nerve conduction velocity, axonal atrophy, paranodal swelling, and demyelination. The addition of myo-inositol to the diet can reverse and prevent this alteration induced by L-fucose (10,38). The chronic administration of myo-inositol ameliorates function and preserves structure in both motor and autonomic models of neuropathy (20,21,36).

The importance of SMIT1 in the sciatic nerve function was further reinforced by the findings of Chau and coworkers, which demonstrated, by using SMIT1 knockout mice, that the level of myo-inositol in the sciatic nerve of adult mice is greatly reduced as well as the nerve conduction velocity (8).

In the present work, we found that the time course for the impact of STZ-diabetes in SMIT1 expression, decreased nerve conduction velocity, and decreased excitability were similar. In addition, the effects were evident after 4 weeks and were maintained at the same level in the other periods. For instance, the decreased nerve conduction velocities of the first and second components of the SN compound action potential occurred at 4 weeks after the onset of diabetes and remained decreased to the same extent in the DB8 and DB12 groups. The first component consists of Aα and Aα/β fibers contributions (30–120 m/s) and the second consists of Aγ fibers (15–30 m/s).

In the SN, in later periods, i.e., 8 and 12 weeks after diabetes induction, the protein level of the second most abundant myo-inositol transporter, HMIT, was also significantly decreased, and since this tissue lacks SMIT2 expression, we predicted that the transport of myo-inositol was likely to be significantly impaired in this tissue. Indeed, the SN has a great proportion of Schwann cells, which are known to have a fundamental role in diabetic neuropathy once the uptake of glucose is higher and the metabolic disturbances are more pronounced in this cell. On the other hand, the DRG soma, when compared to the axonal segment of the SN, for example, has a larger capability to respond to the metabolic insult through a constant resynthesis of proteins. This could explain why the impact of diabetes, probably in the expression of the myo-inositol transporters as well, was more pronounced in the sciatic nerve when compared to the DRG.

To our knowledge, this was the first demonstration of a remarkable decrease in both SMIT1 and HMIT in the SN in STZ-induced experimental diabetes. Despite the fact that we were not able to correlate these changes with myo-inositol concentrations in these tissues, we observed decreased nerve conduction velocity in the same periods that others found decreased levels of myo-inositol using this same experimental model.
